# Special Issue: Selected Papers from the 1st International Electronic Conference on Entomology

**DOI:** 10.3390/insects13100945

**Published:** 2022-10-18

**Authors:** Nickolas G. Kavallieratos

**Affiliations:** Laboratory of Agricultural Zoology and Entomology, Department of Crop Science, Agricultural University of Athens, 75 Iera Odos Str., 11855 Attica, Greece; nick_kaval@aua.gr

The 1st International Electronic Conference on Entomology (1IECE) was held between 1 and 15 July 2021 on the MDPI Sciforum platform organized and funded by the international journal *Insects*. This event provided an opportunity for scientists from around the globe to communicate their most recent research findings in entomology. In the last decade, there has been tremendous development of entomological research, leading to the publication of thousands of important studies. 1IECE aimed to rapidly spread worldwide advances in insect science to the entire scientific community through the publication of proceedings of selected papers in a Special Issue (SI). This SI employed the same eight fields used for topical subdivisions during 1IECE: Systematics and Morphology, Genetics and Genomics, Biology, Behavior and Physiology, Biodiversity, Ecology and Evolution, Pest Management, Forest and Urban Entomology, Medical and Veterinary Entomology, and Apiculture and Pollinators. In total, 21 1IECE presentations have been published in the journal *Insects* through the traditional peer review process. The contributors to this Proceedings SI represent 23 countries from across the globe: Togo, Senegal, Italy, China, Pakistan, Saudi Arabia, Greece, Serbia, Hungary, Poland, Portugal, Australia, Switzerland, Japan, Russia, The Netherlands, Cyprus, USA, Spain, South Africa, France, United Kingdom, and Argentina.

The studies in this SI deal with various interesting aspects of research, i.e., identification of termite species in West Africa [1], characterization of the microbial symbionts of *Ceratitis capitata* (Wiedemann) (Diptera: Tephritidae) populations [2], the contribution of bacterial symbionts to the thermal tolerance of two aphid species *Rhopalosiphum padi* (L.) and *Sitobion avenae* (F.) (Hemiptera: Aphididae) [3], recording the nematode fauna in Greek forests [4], investigation of genetic variability in *Apis mellifera* L. (Hymenoptera: Apidae) from Serbia through microsatellite loci [5], comparison of damage caused to tomato plants by the biocontrol agents *Nesidiocoris tenuis* (Reuter) (Hemiptera: Miridae) and *Dicyphus cerastii* Wagner (Hemiptera: Miridae) [6], evaluation of numerous essential oil-based microemulsions as grain protectants for management of two major stored-product insects, *Tribolium castaneum* (Herbst) (Coleoptera: Tenebrionidae) and *Trogoderma granarium* Everts (Coleoptera: Dermestidae) [7], investigation of several aspects of the life history of the biological control agent *Neoleucopis kartliana* (Tanasijtshuk) (Diptera: Chamaemyiidae) in Greece [8], validation of theoretical models explaining the persistence of mtDNA variation within populations of *Drosophila obscura* Fallén (Diptera: Drosophilidae) [9], description of how thermal conditions, sex, and population origin may affect stress resistance in *Drosophila subobscura* Collin (Diptera: Drosophilidae) [10], the genetic structure of *Corythucha ciliata* (Say) (Hemiptera: Tingidae) based on mitochondrial DNA analysis [11], utilization of ecological/geographical models to evaluate changes in the distribution of *Oedaleus decorus* (Germar) (Orthoptera: Acrididae) [12], investigation of exposure to heavy metals and population origin on the diversity of microbiota and fitness in *Drosophila melanogaster* Meigen (Diptera: Drosophilidae) and *D. subobscura* [13], a database and checklist of alien insects in Greece [14], evaluation of direct and delayed mortality caused by the anthranilic diamide chlorantraniliprole to adults and larvae of *T. castaneum*, adults of *Rhyzopertha dominica* (F.) (Coleoptera: Bostrychidae), adults of *Sitophilus oryzae* (L.) (Coleoptera: Curculionidae) and adults and nymphs of *Acarus siro* L. (Sarcoptiformes: Acaridae) [15], identification of *Pseudococcus jackbeardsleyi* Gimpel and Miller (Hemiptera: Pseudococcidae) and *Maconellicoccus hirsutus* Green (Hemiptera: Pseudococcidae) as potential vectors of cacao mild mosaic virus (CaMMV) [16], defining relationships between coccinellids and aphids on alfalfa in Spain [17], exploration of the diversity and phylogeny of Tingidae species occurring on olive trees in South Africa using morphology and mitogenome sequence [18], and examination of the interaction between *T. castaneum* and *Aspergilus flavus* Link (Eurotiales: Trichocomaceae) in maize flour [19]. Furthermore, this SI includes two review papers addressing the current (2010–2021) global knowledge on taxonomy of Aphidiinae (Braconidae) [20] and adaptation of Echinophthiriidae (Anoplura) surviving in places unfavorable to all other insects [21].

Finally, I would like to thank all authors for their fine contributions, the Academic Editors of Insects Bessem Chouaia, Brian T. Forschler (Editor-in-Chief of *Insects*), Natsumi Kanzak, Silvio Erler, Thomas W. Phillips, Tibor Magura, Marco Salvemini, and David Schlipalius for their critical decisions on certain manuscripts, the reviewers for the time they spent to carefully examine the manuscripts and make valuable suggestions, and the editorial team of *Insects* who processed manuscripts for the SI. My special thanks go to Barbara Wang, Assistant Editor of *Insects*, who exhaustively worked for several months with me to make this important SI come to fruition.
